# Effect of sequential cleavage and blastocyst embryo transfer compared to single cleavage stage embryo transfer on assisted reproductive technology outcome: An RCT

**DOI:** 10.18502/ijrm.v22i6.16793

**Published:** 2024-08-05

**Authors:** Nahid Homayoon, Sahereh Arabian, Esmat Mangoli, Fatemeh Bayati, Maryam Eftekhar

**Affiliations:** ^1^Research and Clinical Center for Infertility, Yazd Reproductive Sciences Institute, Shahid Sadoughi University of Medical Sciences, Yazd, Iran.; ^2^Abnormal Uterine Bleeding Research Center, Semnan University of Medical Sciences, Semnan, Iran.; ^3^Department of Reproductive Biology, Research and Clinical Center for Infertility, Shahid Sadoughi University of Medical Sciences, Yazd, Iran.; ^4^Department of Obstetrics and Gynecology, Clinical Research Development Unit, Hajar Hospital, Shahrekord University of Medical Sciences, Shahrekord, Iran.

**Keywords:** Blastocyst transfer, In vitro fertilization, Embryo transfer.

## Abstract

**Background:**

Assisted reproductive technology (ART), offers hope for many infertile couples by increasing the chance of successful pregnancy. The success of in vitro fertilization depends on various factors, in which embryo transfer (ET) is one of the critical steps influencing in vitro fertilization success rates. Extended embryo culture and blastocyst-stage ET have been considered in ART due to their potential benefits including improved implantation rates.

**Objective:**

This study aimed to compare the outcomes of sequential ET vs single cleavage-stage ET in women undergoing a fresh ET cycle with a limited number of embryos.

**Materials and Methods:**

This randomized clinical trial was conducted on 140 women undergoing infertility treatments and candidates for fresh ET at the Research and Clinical Center for Infertility, Yazd, Iran from August 2023 to January 2024. Women with a number of embryos from 2–5 ( ≥ 2 and ≤ 5 available embryos) were randomly divided into 2 groups: One group underwent sequential ET (one cleavage-stage ET followed by one blastocyst ET) and the other group underwent single-step 2 cleavage-stage ET. The primary outcome was clinical pregnancy, and the secondary outcome included chemical pregnancy and early abortion rates.

**Results:**

Our findings showed significantly higher rates of clinical (33.5% vs 13.6%, p = 0.003) and chemical (41.3% vs 18.2%, p = 0.004) pregnancies in the sequential ET group compared to the single-step cleavage ET group. The early abortion rate was higher in single-step cleavage ET (13% vs 44.4%, p = 0.053).

**Conclusion:**

Sequential fresh ET is a useful choice in women who have a limited number of embryos and can improve ART outcomes.

## 1. Introduction

Infertility is a significant global health issue, affecting a significant number of couples. According to the European Society of Human Reproduction and Embryology fact sheets, one in 6 couples globally experience infertility challenges at least once during their reproductive lifetime. Global data reported a 19% delivery rate from assisted reproductive technology (ART) treatment per ART cycle and a 30.7% cumulative delivery rate (1).

Numerous factors can impact the outcome of in vitro fertilization (IVF) and play a crucial role in determining its success. One of the most important and challenging steps is embryo transfer (ET) and successful implantation. The assessment of ET involves a comprehensive examination from various perspectives. Factors such as whether the embryo is fresh or frozen, the embryonic stage (cleavage or blastocyst), the quality of the transferred embryo, and the condition of the endometrium during the ET process (2–4).

The widespread adoption of extended embryo culture represents a notable trend in ART. Advocates of this approach believe that the blastocyst ET enhances reproductive outcomes by reducing aneuploidy rates and better synchrony with the uterus. Recently, studies focusing on women with favorable prognoses, present evidence suggesting an increased likelihood of achieving live birth after fresh blastocysts transfer in comparison to cleavage-stage embryos (5, 6).

According to a recent Cochrane review, 27 randomized controlled trials analyzed. The findings indicated that the live birth rate in the fresh blastocyst transfer group was higher than in the fresh cleavage-stage ET group (6).

Despite all the mentioned benefits of blastocyst transfer, there is always concern about the cancellation of the blastocyst-stage ET cycle due to lack of cleavage-stage embryo development to the blastocyst stage. Despite observations of adequate development of embryos in vitro on days 2–3, the lack of predicting indicators for blastocyst development increases the risk of having no embryos to transfer (7).

Due to concerns about the potential unavailability of embryos for transfer, particularly in individuals with a restricted number of embryos, our study chose the strategy of sequential fresh ETs. This approach was performed in women with 2–5 embryos in the cleavage stage to reduce the concern about a scenario where no transfer is possible in this group. Additionally, we could use the benefits of blastocyst ET by using this method.

This study aimed to assess and compare the ART outcomes between the single-step 2 cleavage ET group and a sequential ET group.

## 2. Materials and Methods

### Participants and trial design 

This randomized clinical trial study comparing 2 methods of fresh ET was conducted at the Research and Clinical Center for Infertility, Yazd Reproductive Sciences Institute affiliated to the Shahid Sadoughi University of Medical Sciences, Yazd, Iran. Women receiving infertility treatment, and candidates for fresh ET randomly divided into 2 equal groups from August 2023 to January 2024.

### Ovarian stimulation, oocyte puncture, and ET

All women underwent the antagonist protocol for controlled ovarian stimulation. Stimulation started on day 2 of the menstrual cycle with follicle-stimulating hormone and 150–300 IU of recombinant (Cinnal-F, CinnaGen, Tehran, Iran) and human menopausal gonadotropins (Humegnan, DarouPakhsh, Karaj, Iran) according to the individual's age, antral follicle count and anti-Mullerian hormone levels. Gonadotropin-releasing hormone antagonist was added when at least one follicle reached a diameter of ≥ 14 mm, and we used 0/25 mg Cetrorelix Acetate (Cetroronax, Ronak, Tehran, Iran).

For dual triggering, 1 mg of Buserelin Acetate (CinnaFact, CinnaGen, Tehran, Iran) and 5000 IU human chorionic gonadotropin (Folignan, DarouPakhsh, Karaj, IRAN) were used when ≥ 2 follicles reached a size of ≥ 17 mm.

Oocyte retrieval was performed 34–36 hr after triggering. IVF or intra cytoplasmin sperm injection was performed and 2 days later, after determining the number of embryos for each person, those with a number of embryos from 2–5 ( ≥ 2 and ≤ 5 available embryos) and candidates for fresh ET were randomly divided into 2 groups. One group underwent sequential ET (one cleavage-stage ET followed by one blastocyst ET) and the other group underwent single-step 2 cleavage-stage ET.

IVF/intra cytoplasmic sperm injection candidates aged 18–45 yr due to infertility caused by various factors such as diminished ovarian reserve, male factors, ovulation and tubal disorders, endometriosis, and unexplained infertility were included in the study. Women with anatomical uterine abnormalities and uterine myoma larger than 40 mm were excluded.

The culture media for all women was SAGE 1-step, Cooper Surgical, Denmark. All transfers were conducted using the RADA ET catheter (Behrad Rooyesh Rooyan Company, Iran).

Luteal phase support started on the day of oocyte retrieval and all women received both oral progesterone (dydrogesterone) 10 mg twice a day, along with 400 mg of vaginal/rectal progesterone twice daily.

### Outcome measure

The primary outcome was clinical pregnancy, while the secondary outcomes were chemical pregnancy and early abortion rates. A positive chemical pregnancy was identified 12 days after ET if the serum beta-human chorionic gonadotropin level was > 50 IU/L. The observation of fetal heart activity 2 wk after the positive beta-human chorionic gonadotropin is considered a positive clinical pregnancy. Early abortion was defined as loss of gestational sac or fetal heartbeat in clinically pregnant women till 8 wk of gestational age.

### Sample size

The minimum sample size was estimated to be 70 in each group by considering the significance level of 95%, a power of 80%, along with a 20% difference between groups, based on a similar study (8).

### Randomization

The randomization list of samples was prepared using the random allocation1 software. Subsequently, according to the order referral of participants and inclusion criteria, the statistical consultant informs the researcher about the assignment based on the participant's entry number from the list. Each sample assigned to the intervention or the control group according to its assigned study group.

### Ethical considerations

The present study was approved by the Ethics Committee of Yazd Research and Clinical Center for Infertility, Shahid Sadoughi University of Medical Sciences, Yazd, Iran (Code: IR.SSU.RSI.REC.1402.008). The study subsequently registered with the Iranian Registry of Clinical Trials on August 27, 2023. The last update on the Iranian Registry of Clinical Trials website made on March 30, 2024. Written inform consent obtained from all participants.

### Statistical analysis

The statistical analysis was performed using SPSS software (Statistical Package for the Social Sciences, version 26.0, Chicago, IL, USA). Continuous variables were presented as means ± standard deviations, while categorical data were expressed as frequencies (%). The comparison of continuous variables between research groups was performed using the Mann-Whitney test and the *t* test, and categorical variables were assessed using the Chi-square test. For each test, a 2-tailed p < 0.05 is considered statistically significant.

## 3. Results

**Figure 1:**
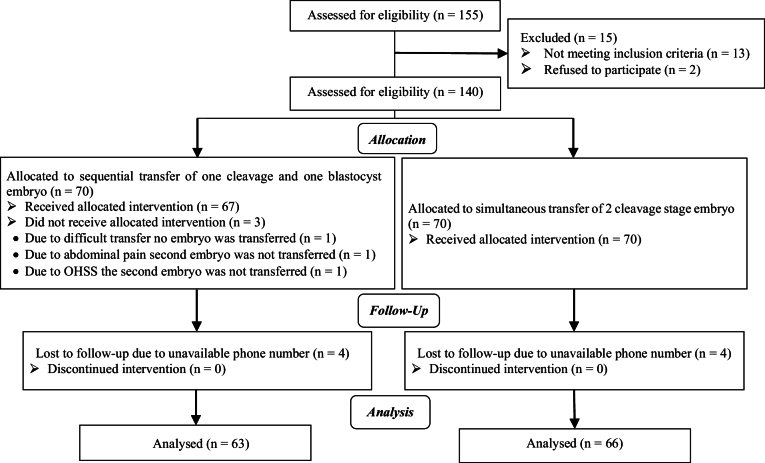
CONSORT flowchart. OHSS: Ovarian hyperstimulation syndrome.

A total of 140 women who met the inclusion criteria, were randomly divided into 2 equal groups (n = 70). 3 cases in the sequential group were excluded due to interruption of the ET caused by ovarian hyperstimulation syndrome, abdominal pain, and difficult transfer. Additionally, 5 cases in the sequential group and 4 cases in the single-step ET group were excluded during telephone follow-up due to incorrect telephone numbers. Ultimately, 128 cases were included in the study. The CONSORT flow chart and trial design are illustrated in figure 1.

There was no significant difference in the demographic and clinical characteristics between the groups. The mean age, body mass index, duration and type of infertility, anti-Mullerian hormone levels, as well as the number of retrieved oocytes and high-grade embryos on day 2/3, were compared between groups. Groups were similar in terms of recurrent pregnancy loss and/or recurrent implantation failure (RIF) (Table I).

The pregnancy outcomes in the sequential cleavage and blastocyst ET group exhibited a significant difference compared to cleavage stage ET (Table II). The primary outcome and chemical pregnancy were 36.5% in the sequential ET group, which was significantly higher than the 13.6% observed in the cleavage ET group (p = 0.003). Secondary outcomes also showed a statistically significant difference in chemical pregnancy (41.3% vs 18.2%, p = 0.004). Although early abortion rates were higher in the cleavage stage ET compared to the sequential ET group. This difference did not reach statistical significance (13% vs 44.4%, p = 0.053).

In the sequential ET group, 14 cases were unable to proceed to the second step of ET due to the absence of the obtained blastocyst. Table III shows a comparison between the 2 groups after excluding these cases. The results indicated that clinical and chemical pregnancy rates were significantly higher in the sequential ET group (p = 0.002 in clinical pregnancy and p = 0.007 in chemical pregnancy). One case in this group developed ectopic pregnancy.

**Table 1:** Demographic and clinical characteristics of study groups


**Variable**		**Sequential cleavage and blastocyst transfer (n = 63)**	**One step cleavage transfer (n = 66)**	**P-value**
Age (yr) ${\;}^{*}$		34.03 ± 5.48	33.59 ± 6.09	0.604
BMI (kg/m^2^) ${\;}^{*}$		27.07 ± 5.16	26.6 ± 4.52	0.667
AMH (ng/ml) ${\;}^{**}$		2.44 ± 2.36 (1.80, 2.2)	2.38 ± 1.94 (1.80, 2.1)	0.756
Duration of infertility (yr) ${\;}^{**}$		6.40 ± 5.05 (5.00, 8.00)	6.06 ± 4.02 (8.00, 5.00)	0.934
Type of infertility ${\;}^{***}$				
	**Primary**	34 (54)	39 (59.1)	
	**Secondary**	29 (46)	27 (40.9)	0.557
Cause of infertility ${\;}^{***}$				
	**DOR**	15 (23.8)	17 (25.8)	
	**Male factor**	16 (25.4)	21 (31.8)	
	**Mix**	10 (15.9)	10 (15.2)	
	**Unexplained**	20 (31.7)	16 (24.2)	
	**PCOS**	2 (3.2)	1 (1.5)	
	**Endometriosis**	0 (0)	1 (1.5)	0.775
Number of retrieved oocytes ${\;}^{**}$		7.48 ± 3.62 (7.00, 5.00)	8.50 ± 3.26 (8.00, 5.00)		0.031
Number of produced embryos ${\;}^{**}$		3.71 ± 1.18 (4.00, 2.00)	3.35 ± 1.18 (3.00, 2.00)		0.092
History of RIF and/or RPL ${\;}^{***}$		11 (17.5)	11 (16.7)		0.905
*Data presented as Mean ± SD, Student's *t* test. **Data presented as Mean ± SD (Median, IQR), Mann-Whitney test. ***Data presented as n (%), Chi-square test. AMH: Anti-Mullerian hormone, BMI: Body mass index, PCOS: Polycystic ovarian syndrome, DOR: Diminished ovarian reserve, RIF: Recurrent implantation failure, RPL: Recurrent pregnancy loss				

**Table 2:** ART outcome between groups


**Variable**	**Sequential cleavage and blastocyst transfer (n = 63)**	**One step cleavage transfer (n = 66)**	**P-value**
**Chemical pregnancy**	26 (41.3)	12 (18.2)	0.004
**Clinical pregnancy**	23 (36.5)	9 (13.6)	0.003
**Early abortion**	3/23 (13)	4/9 (44.4)	0.053
Data presented as number (%), Chi-square test. ART: Assisted reproductive technology			

**Table 3:** ART outcome between groups, excluding cases with no blastocysts


**Variable**	**Sequential cleavage and blastocyst transfer (n = 49)**	**One step cleavage transfer (n = 66)**	**P-value**
**Chemical pregnancy**	20 (40.8)	12 (18.2)	0.007
**Clinical pregnancy**	19 (38.8)	9 (13.6)	0.002
**Early abortion**	3/19 (15.8)	4/9 (44.4)	0.165
Data presented as number (%), Chi-square test. ART: Assisted reproductive technology			

## 4. Discussion

The findings of our study suggested that in the fresh ET cycle, sequential transfer of 2 embryos improved chemical and clinical pregnancy outcomes compared to a single-step transfer of 2 cleavage-stage embryos, in women with a limited number of available embryos.

The first time that sequential ET was presented, the authors concluded that 2-step ET can be beneficial to improve the pregnancy rate in IVF-ET cycles without freezing facilities (9). Subsequently, numerous studies in literature have reported similar or contrasting outcomes.

In line with our results, a retrospective cohort study showed that sequential ET is an effective option for poor ovarian response women, and the live birth rate in the sequential transfer group was significantly higher compared to the cleavage ET group and demonstrated similarity to the blastocyst ET group (8). This shows that sequential ET is as efficient as 2 blastocyst transfer. An RCT showed that in women with RIF, treatment with, the sequential ET approach resulted in significantly enhanced pregnancy outcomes compared to regular day 3 transfers (4). Similar to the previous study, a systematic review showed that sequential ET improves clinical pregnancy rate compared to conventional cleavage-stage ET in both RIF and non-RIF subgroups (10). Another study in frozen ET cycles in women with RIF found that the success rates in the sequential ET group were significantly higher than those in the day 3 cleavage-stage ET group, and were similar to those in the blastocyst transfer group (11). A prospective and RCT showed that within IVF fresh cycles, sequential ET led to notably higher rates of implantation and clinical pregnancy compared to either cleavage transfer on day 3 or blastocyst transfer on day 5 (12).

Contrarily, some conflicting data in the literature indicate no discernible benefit to sequential ET. An RCT revealed that the implementation of a double ET did not increase pregnancy rates compared to blastocyst ET on day 5 in individuals experiencing 3 or more implantation failures. Similarly, a retrospective study concluded that sequential ET did not demonstrate superiority over single-step cleavage or blastocyst ET in cases with RIF (13, 14).

Several mechanisms may contribute to, the observed, higher success rates associated with 2-step or sequential ET. One possible explanation is the documented higher implantation rates for blastocysts found in the literature. This elevated success may be attributed to various factors, including molecular mechanisms and synchronous coordination of embryo development and endometrial receptivity (15–17).

An additional potential mechanism contributing to the increased success rates in sequential ET could be the favorable impact of the first embryo on the implantation of subsequently transferred blastocysts. The cytokines produced by the initial ET catheter, as well as by the first embryo and endometrium may have a positive effect that could extend to the second transferred embryo. This cascade of cytokine release might provide an environment conducive to improved conditions for the implantation of the subsequent embryo during the second transfer (18, 19).

Sequential ET can reduce the risk of ET cycle cancellation as compared to double blastocyst transfer, since extended culture may result in an insufficient supply of blastocysts for transfer.

Our study's strength lies in the strategic transfer of blastocysts for women with a limited number of embryos in a fresh ET cycle.

One limitation of our study was our center's policy to freeze embryos for cleavage stage transfers. Consequently, for women in the sequential transfer group with 4 or 5 embryos, only 2 were chosen for extended culture and blastocyst formation, the remaining embryos were frozen as cleavage. If all embryos had been utilized for extended culture, the number of cases with unavailable blastocysts for the second transfer in the sequential ET group might have been reduced.

We propose the design of another RCT with 2 groups, a sequential transfer group and a blastocyst transfer group of eligible women for fresh ET.

## 5. Conclusion

Sequential fresh ET is a useful choice in women who have a limited number of embryos and can improve ART outcomes.

##  Data availability

Data supporting the findings of this study are available upon reasonable request from the corresponding author.

##  Author contributions

M.E.: Study design and protocol. M.E., N.H., S.A., F.B.: Conducted the procedures and data analysis. E.M. was responsible for embryologic procedures. Every author took part in the literature review, helped in drafting the manuscript, gave their approval to the finished version of the manuscript, and assumed accountability for the data's integrity.

##  Conflict of Interest

The authors declare that there is no conflict of interest.
